# Hospital and Institutional News

**Published:** 1917-09-01

**Authors:** 


### HOSPITAL AND INSTITUTIONAL NEWS.

### ORTHOPÆDICS IN THE WEST RIDING.

THE completion of the extension of the 2nd Northern General Hospital at Beckett's Park will be marked by the visit of the American Ambassador to Leeds in October. Mr. Charles Lupton, so well known in connection with the Leeds General Infirmary, inaugurated the scheme at the beginning of his mayoralty in 1915. It provides, at a cost of £25,000, for 720 additional beds, and worthily co-ordinates the work which makes the institution the centre of orthopædics in the Northern Command. King Manoel and other visitors have seen and assisted in its development, and the extensive mechanical equipment is completed by the curative workshops, where curative training is practised on a large scale. The whole West Riding has generously supported the scheme, which is another triumph for Mr. Lupton's initiative and example.

### AMERICAN WAR HOSPITALS.

AMERICA is taking a leaf out of Canada's book in the matter of organising war hospitals. A number of leading American medical men, representing the most important hospitals in New York, Boston, and Philadelphia—all of them members of the American Red Cross Society—have just completed a tour of the institutions of the Canadian Military Hospitals Commission. The object of their visits was to glean first-hand information as to the methods and treatment adopted in dealing with returned wounded soldiers, a problem to which Canada has addressed itself with characteristic thoroughness. The United States are likely to follow in the footsteps of the Dominion in this respect.

### THE WOUNDED IN FRANCE AND THEIR FRIENDS.

MOST workers at home, in proportion to the pressure upon each, have moments when they feel the strain of war to repletion. A good antidote is to try to realise the added strain on hospital workers in France near the Front, where the work is onerous, severe, and full of anxiety, whilst the weight of it is increased by the ever-recurring arrival of new and yet more urgent cases, demanding immediate and continuous attention. Mr. David Pendrigh, a grateful father, bears the following testimony in the *Times* of the 24th instant:—

SIR,—Having just had the mournful privilege of visiting an army hospital in France, I feel that I must say a word in public about the care of the wounded over there. It is the only way in which I can discharge the debt of gratitude I owe to the noble band of workers—the colonel commanding the hospital, the matron, the sisters, and the orderlies—who fought so untiringly and devotedly to save my wounded son from death, and who treated me personally with a sympathy and a kindness which I cannot adequately repay. In writing this I know I am expressing the sentiments of the party of anxious relations of wounded whom I met under the hospitable roof of a British Red Cross hostel at a certain base. To all of us the perfection of the medical and nursing arrangements, and the more than courtesy, the loving care, lavished upon us, were a revelation. No Royal personages could have their way into and through France made smoother for them than visitors to the wounded. I would especially thank the Red Cross and V.A.D. young ladies who, without fee or thought of reward, conduct the hostels for relations, and whose cheerful solicitude lightens the days, in some cases weeks, of nerve-racking anxiety endured by those who have, in the official phrase, been "permitted to visit" the wounded. The cost of all this organisation cannot be realised by those who have not seen the system at work, and if these remarks of mine help to increase the flow of money into the coffers of the British Red Cross, I trust I shall be excused for rushing into print.—Yours truly,

DAVID C. PENDRIGH.

Reuter's, 24 Old Jewry, E.C., Aug. 23, 1917.

We echo the hope expressed, and believe this grateful testimony to a splendid work and the workers will yield much fruit in material benefit to the Joint War Committee, and in comfort and cheer to all, who have the privilege to minister in the war hospitals in France and elsewhere.

### A WARD ON WHEELS.

EVEN the modern motor ambulance cannot be said to have met all the needs of the war. The serious cases, so far as possible, have often to be kept in the clearing stations, and then, after waiting, to undergo the strain of a train journey, which even in hospital trains it is often a risk to take. The serious case requires special conditions of comfort, temperature, and silence, for which the new types of ambulance have now been designed by the motor ambulance department of the Joint War Committee. One type is a single-bedded ambulance, with a light body, folding doors, and an electric heating installation. It is made so that a patient may be carried in it in comparative comfort for a considerable distance. The second is for two beds, in form similar to berths, fitted with electric warmers, and built with duplex panels, with an insulating medium between, so as to make them sound-proof. Heating-pipes in parallel rows under each bed are warmed to the desired degree by admitting a part or all of the exhaust gases from the engine. The windows are also duplex, and the ventilation appears to be artificial. Trials oversea are being made to test their value, when it will be seen if they enable serious cases to be removed by road under such conditions of quiet and temperature as are essential to their comfort. The new types are more like wards on wheels than anything which has yet been attempted in a motor ambulance.

### WELL-KNOWN LEICESTER SURGEONS HONOURED.

IT is a source of considerable satisfaction to find the names of two well-known contemporary Leicester surgeons appearing almost simultaneously in the recent honours lists. Colonel C. J. Bond is vice-chairman of the board of the Leicester Royal Infirmary and a consulting surgeon to that institution. As a member of the Medical Research Committee and also in the capacity of honorary consulting surgeon to the Midland military area he has performed notable public service. He is well known as the contributor of many important articles to the medical press on original research, and his name is frequently quoted in current literature bearing upon research work. His name has twice appeared in the list of the Secretary of State for War as performing meritorious service, and he is now awarded the C.M.G. Mr. G. C. Franklin, another consulting surgeon to the Leicester Infirmary, whom Colonel Bond succeeded as senior surgeon, is awarded the membership of the Order of the British Empire for his services as medical officer of the Hawkstone Red Cross Hospital at Fareham, Hants. He was president of the British Medical Association at the Leicester Conference some years ago, and at the succeeding annual meeting of the Association at Toronto he was awarded the Honorary LL.D. of Toronto University. The promotion to Brevet-Colonel of Lieut.-Colonel L. K. Harrison, R.A.M.C. (T.), the Administrator of the 5th Northern General Hospital at Leicester, is also of interest.

### PATIENTS' PREJUDICES.

The war-time expenditure of Walsall Hospital has created a difficulty which has caused two interesting suggestions to be made. One refers to a new source of income, the other to a prejudice on the part of patients which is now being investigated. The first is that the employees in the different factories should follow the example of the workmen in Birmingham and maintain certain beds in the institution. Time and trouble should make this possible. The second suggestion is that a prejudice on the part of working men in Darlaston against the Walsall Hospital should be investigated. It appears that while most of the serious accident cases in Darlaston are taken to Walsall, yet the working classes there display a marked preference for the Wolverhampton Hospital. This is curious, and we are glad that the hospital has had the courage to face the fact, and openly to set to work to find the explanation. The reason once known, the prejudice can be removed in a little time.

### WORKMEN'S REPRESENTATION AT MERTHYR.

As we prophesied at the time, the closing of a number of beds in Merthyr General Hospital has provided no solution for its financial difficulties. The board now hopes to enlist the workmen's co-operation by certain recommendations. These, which are being submitted to mass meetings of the workmen, agree that societies or small bodies of workmen which subscribe £5 a year shall be entitled to nominate a governor; that, while the position of vice-presidents remains unchanged, a new vice-president shall not be entitled to a seat on the executive board except by special resolution; and that the elected members of the executive should number twenty-two instead of fifteen. The number of workmen shall be increased from six to twelve, of employers from three to four, while six shall be elected by the governors. The board also recommends that both employers and workmen shall be allowed to appoint their own representatives so long as employers maintain a subscription of £50 a year and workmen give 4s. a head annually. Neither employers nor workmen will as governors take part in the election of the six members chosen by the governors. It will be seen that the representation of the workmen is considerably increased under the new scheme, and if this change is really welcomed by the men the necessary funds should quickly be found to re-open beds, the closing of which has for the hundredth time proved to be no solution of the difficulty.

### BRIDGEND MILITARY HOSPITAL.

Bridgend Union Infirmary is to be taken over as a military hospital, and at the last meeting of the board the question of its staffing was discussed. Colonel W. Coates, Assistant Director of Medical Services, Western Command, wrote suggesting that the staff should consist of two sisters, four or five fully-trained nurses, and five or six probationers, while two porters, who might be discharged soldiers, would suffice in place of the five R.A.M.C. and five working orderlies proposed by the Guardians. The board adopted these suggestions, and fixed the salary of Dr. W. Edmund Thomas, subject to War Office approval, at £105 per annum.

### MACCLESFIELD INFIRMARY AND ANONYMOUS CRITICS.

It is not often that anonymous critics can create as much stir as they have done among the staff of Macclesfield Infirmary. A deputation of nurses has been heard by the board, and a rota of house visitors has been established. It is admitted that one of the nurses had committed "an error of forgetfulness," but the staff resented allegations that its members did not work amicably together, that the matron did not treat them courteously, and that the food provided for them was deficient and unappetising. Alderman Frost lately declared that the committee of the infirmary had been fighting against all sorts of innuendoes, though no specific charge had been made. The mischief caused has probably been cleared away by this public discussion within the institution. It is fairly clear, however, that the anonymous critics would not have been able to cause the trouble which they have if one nurse had not been proved at fault. The whole staff has suffered; for a hospital nurse must be above suspicion.

### THE PENSIONS APPEAL TRIBUNAL.

Judge Parry, the chairman of the Pensions Appeal Tribunal, took advantage of the first decision promulgated to explain its origin and duties. Originally, he explained, the granting of a pension was decided solely on medical grounds. Now it has been decided that the man himself should have the right to support his case before a pension was refused. A man's statement, whether supported by medical evidence or not, was essential to a decision of his claim, since his statements might be correct in fact, while the evidence recorded in the military medical documents might be inadequate. Even now, he thought, the wording of the forms used might be drafted more in the spirit of the new warrants. A Tribunal was essential to help the Pensions Ministry in doubtful cases, and the decision of the Appeal Tribunal was to be final. Its procedure was designed so that it might have neither the form nor the spirit of litigation. The new warrants of March last were an improvement. It remains with the Appeal Tribunal to see that pensions are granted in all cases which, considered liberally, come within their scope.

### THE BUSINESS SIDE OF CRIPPLES' WORKSHOPS.

THE training schools and workshops for disabled soldiers have been sufficiently long in existence for an idea to be gained of them as business concerns. The Lord Roberts Workshops in Fulham Road, where 800 men, assisted by their female relatives in light work, are employed, realised a net profit of £965 during the past year. This was spent in purchasing the lease of a house next door, where the men can receive the special treatment suited to their condition. A large number of firms act as sale agents, and the goods themselves are not only well made, but sold at fair market prices. Wages begin at £1 a week, and rise in a few cases to £2 10s. or even more. In addition, each man receives his pension irrespective of his earnings. Large orders are received from different firms, and contracts are also made with such bodies as the War Office Canteen Committee. Skilled men are engaged on the production of the workshop catalogues; posters are designed and executed for theatres, and some of the processes are completed at branch workshops in the provinces. The training is valuable not only in its effect on the man himself, but also because it allows him to work under careful supervision, so that he does not lose heart, as he might do, if he had to fight his own way alone. At the same time a man is free to seek another employer, and, what is more, to return to the workshops if the change has not proved a success. This side of the work deserves to be as well known as the skill which the men achieve under careful training.

### COUNTY VISITATION SOCIETY.

THROUGH the County Folk Visitation Society, of which Colonel E. T. Clifford is chairman, an effort is being made to organise on a territorial principle the visitation of wounded soldiers in London hospitals whose relatives and friends are too far away to pay frequent visits. The aim is to bring a touch of home to the bedsides of these men, in the shape of some friend who knows their native town or village and who is able to talk of the patient's native heath. A scheme of this sort has been in operation for some time under the auspices of the Cumberland and Westmorland Association of London. A band of lady visitors has already paid over 600 visits to Lakeland soldiers in forty-three hospitals in the London area, taking with them comforts such as are appreciated by the men. Another branch of the work undertaken by this Association has been the provision of funds to assist parents and wives to journey from Lakeland to see their sons and husbands in hospital, and also to enable patients discharged from hospital to proceed home.

### ADDENBROOKE'S HOSPITAL, CAMBRIDGE.

WE wonder why the committee of management of Addenbrooke's Hospital does not take the plunge and bring the accounts into uniformity with those of the vast majority of the hospitals of the country. As we have suggested to some of the comparatively few hospitals that are behind the times in this respect, now is a favourable moment to make an alteration. There is something unsatisfactory in examining an income and expenditure account for twelve months ending September 30, an account which is not comparable with any other hospital, and, therefore, unprogressive, to use an adjective which cannot be applied to any other part of the work of the great Cambridge hospital. Perhaps the committee will take advantage of the world being upside down and make a much-to-be-recommended change preparatory to the happy hour when we shall all go back to a peaceful routine. We notice that the executive does not believe in the abridged report, and presents the subscribers with 170 thick pages, not counting a number of excellent illustrations. In this action they conform with the practice of most hospitals. There are very few indeed that are seriously abridged, although here and there a few pages of unessential statistics are found missing.

### FIRST-HAND INFORMATION FOR FRIENDLY SOCIETIES.

FOR many years Addenbrooke's Hospital, Cambridge, has received valuable support from the various Friendly Societies' Hospital Sunday Parade Committees throughout the district. In 1915 many of these parade collections reached a record amount. The secretary-superintendent, Mr. R. J. Coles, conscious of the need of increased support for the hospital, in 1916 offered to attend the various Hospital Sunday Parades and address the meetings, generally in the open air, on the work and needs of the hospital. The result was that the hospital received the sum of £405 over and above the record collections of 1915. This work is being continued during the present year, and so far there is every reason to believe that the income received from this source will be considerably larger than the welcome sum received last year. A few of the individual collections may be mentioned. At Littleport in 1916 the sum of £178 was raised, of which Addenbrooke's received £154. This year the sum was £250 for the benefit of Addenbrooke's and several other institutions. At St. Ives, Huntingdonshire, where the Hospital Sunday Parade had been allowed to lapse for several years, the sum of £62 was raised, £31 being received by Addenbrooke's and £31 by the County Hospital, Huntingdon. At Southery, a village of some 500 inhabitants, in 1915 the Parade Committee raised the sum of about £4, in 1916 about £9, and this year they have raised and sent to Addenbrooke's the handsome sum of £43. The foregoing proves that the people desire to receive information about their hospital first-hand, and appreciate the fact that someone from the institution takes the trouble to attend their annual parades, and personally to thank them for the support which they give to the institution.

### THE DETERMINATION OF SEX.

It would assuredly be of the very greatest value to be able to decide the sex of an offspring before its birth. Even when we consider only the lower animals, everyone can understand the importance of being able to determine whether the calf or lamb should be male or female; for instance, in a herd of dairy cows the farmer would be glad indeed if he could arrange that a large proportion of the calves should be females. If it is admittedly important in the case of the lower animals, it is, at least, no less important in the human race. To put it merely on a money basis, it is not rarely of the greatest moment to the members of a family that a male child should be born, and, therefore, any method which claims to have solved this problem is worthy of careful consideration. Again, it is no rare occurrence for a family of children to consist of a long series of boys without a single girl, and it is also not at all rare to find in one family several girls and not a single boy. Parents would often give much to be able to have a child of the sex they wish. One of the latest claims to the power of determining the sex of the child is contained in a work by Dr. Rumley Dawson. In this book he brings forward evidence which, in his opinion, proves that in the human female all the ova from one ovary are destined to produce male infants, and all the ova from the other ovary can give rise only to female children; and he claims, by the application of his rule, to have accounted for the sex of a child in 97 per cent. of the cases. The first argument which presents itself against this theory is found in the fact that the author limits his theory to the human being. Now, to put it at the lowest level, it is intensely unlikely that the determination of sex should be absolutely different in human beings from what it is in other mammalia. Certainly no such rule can hold in birds, where there is an ovary only on one side, and we know that in some of the invertebrates the conditions of the impregnation of the ovum may determine the sex of the animal. Further, it has been shown that if caterpillars are starved before entering the chrysalis state, the butterflies or moths into which they develop are males, while if others of the very same brood receive ample nourishment they develop into females. It may be said that the conditions which determine sex may be utterly different in the insect and in man, and this must be acknowledged, but it is much more difficult to believe that the method by which sex is determined in man is utterly different from that obtaining in the other mammalia. Perhaps the most easily comprehensible argument against the ovary theory of sex is to be found in the results of ovariotomy. There may be cases, it is true, in which children of only one sex are born after removal of one ovary, but it is by no means the rule, and the exceptions cannot be glossed over by saying that in the exceptions portions of the ovary must have been left behind. No! The mystery of sex has not yet been solved. We cannot doubt that one day the true reasons of sex will be known, but it will not hasten that day to accept theories, however specious, which rest on no secure foundation of facts.

### THE WELSH NATIONAL MEMORIAL ASSOCIATION.

The Pembrokeshire Authority is not the only one which has fallen foul of the Welsh National Memorial Association. At the last meeting of the Newport Corporation there was a long discussion, and an amendment to delay a decision was rejected. Ultimately it was resolved that no further financial aid shall be afforded to the Association, beyond the contributions to which the Council already stand committed, until Newport obtains what it regards as satisfactory representation on the governing body of the Association. In explaining why Newport was standing out of the scheme in this way, Mr. Robjent, chairman of the Finance Committee, said it was not a matter of questioning the great good the Association was doing in its efforts to combat consumption, but of getting rid of financial inefficiency in the administration.

### THIS WEEK'S DRUG MARKET.

The advancing tendency of prices continues, and there seems to be no prospect of any alteration in this tendency in the near future. This week's heavy storms have no doubt done considerable damage to the peppermint and other medicinal herb crops which have not yet been harvested, and at the best the yield of English peppermint oil can only be poor. Among drugs that are again dearer are star aniseed oil, cassia oil, cream of tartar, guaiacum, menthol, and opium. The last-named is scarce, and the advance in price is substantial, with the probability that the highest figure has not yet been reached. The value of camphor is firmly maintained. Higher prices are asked for gentian root. Liquorice juice is very scarce, and prices are advancing. There is no reduction in the high price of permanganate of potash. The large demand for saccharin continues, but can only be met very inadequately. Honey is in plentiful supply, and prices have a lower tendency. Business has been done in gum acacia at rather high rates. Squill is lower in price. The value of citric acid is not quite so firmly maintained, and the same may be said of tartaric acid.

### TO OUR READERS.

Contributions are specially invited from any of our readers to these columns. They should deal with topical subjects and news. They must be authenticated for the information of the Editor only. The minimum payment if published is 5s. There is no hard-and-fast rule as to space, but notes of about twenty lines in length are preferred.

